# Supplementary material for: Implementing a self-monitoring application during pregnancy and postpartum for rural and underserved women: A qualitative needs assessment study
Source: PLoS One. 2022 Jul 19;17(7):e0270190. doi: 10.1371/journal.pone.0270190 (PMC9295984; doi:10.1371/journal.pone.0270190)
Supplement: S1 File — (DOCX) [file pone.0270190.s001.docx]

Vida Interview Questions – Health Care providers

Pregnancy Care Experience:

1. What month did your patients typically first access prenatal care?

2. How easy is it for the women you care for to talk to a nurse when she has questions?

3. Moms report difficulty returning for their 6 week postpartum visit. Would you be wiling to do follow up visits via a video visit? Why or why not?

Describe Vida App

This project is developing a way for women and their physicians to monitor blood pressure, weight, and mood during pregnancy. Mothers will take their blood pressure and weight at home with a simple electronic device that is provided by their insurance and text it to the Vida app daily. Once a week, she will answer a question about her mood. If a mother’s blood pressure or weight are too high, she will be sent a text to contact her physician, as it may indicate that she is experiencing a dangerous health condition. If mom’s mood has been sad for two weeks or more, she will also be sent a message with information about resources and instructions to contact her physician. Both moms and their health care providers will have access to their data through a password protected website. Monitoring women during pregnancy and after birth may identify when they are at risk of developing serious complications. Eventually we will be able to integrate FitBit activity and sleep behavior into the data, allowing mom’s to their data.

We are working with ACOG and HRSA representatives who are interested in reducing barriers to care and amplifying the care women are able to access. This proposed remote monitoring app intends to help providers identify women at risk earlier in their pregnancies.

1. Would you be willing to participate in a monitoring program like this if it were available? Why or Why not?

2. What would make this better?

3. What would you like to get out of a monitoring program like this?

4. Would you be willing to receive notifications about the women’s health in between visits?
