# Supplementary material for: Implementing a self-monitoring application during pregnancy and postpartum for rural and underserved women: A qualitative needs assessment study
Source: PLoS One. 2022 Jul 19;17(7):e0270190. doi: 10.1371/journal.pone.0270190 (PMC9295984; doi:10.1371/journal.pone.0270190)
Supplement: S2 File — (DOCX) [file pone.0270190.s002.docx]

Focus Group Questions: Mothers

Ice Breaker: How many children does everyone have?

Pregnancy Care Experience:

1. What month did you first access prenatal care in your last pregnancy? Was that earlier or later than other pregnancies?

2. Did you receive any referrals for special care or follow up – if so, were you able to access care?

3. Did you have trouble accessing regular care during pregnancy, why or why not? Were you able to talk to a nurse or physician when you had questions?

4. Did you go to your 6 week follow up appointment after birth? Why or why not? Would having access to a virtual appointment with your doctor make that visit easier?

Technology Experiences

1. Do you have a cell phone and is it a smart phone?

- How often do you use it to text?
- Do you have access to data on your cell phone?
- How often do you change your cell phone plan and phone number?

2. Do you have internet access in your home?

- How comfortable are you accessing WiFi in your home, if you are able?
- Can you or someone in your household troubleshoot internet problems?

Describe Vida App

This project is developing a way for women and their physicians to monitor blood pressure, weight, and mood during pregnancy. Mothers will take their blood pressure and weight at home with a simple electronic device that is provided by their insurance and text it to the Vida app daily. Once a week, she will answer a question about her mood. If a mother’s blood pressure or weight are too high, she will be sent a text to contact her physician, as it may indicate that she is experiencing a dangerous health condition. If mom’s mood has been sad for two weeks or more, she will also be sent a message with information about resources and instructions to contact her physician. Both moms and their health care providers will have access to their data through a password protected website. Monitoring women during pregnancy and after birth may identify when they are at risk of developing serious complications. Eventually we will be able to integrate FitBit activity and sleep behavior into the data, allowing mom’s to their data.

1. Would you be willing to participate in a monitoring program like this if it were available?

2. What would make this better?

3. What would you like to get out of a monitoring program like this?

4. Do you think your physician would like to get notifications about your health in between visits? Would you like him or her to be able to see your daily readings, if you were at risk of complications during pregnancy?
